# Supplementary material for: High accuracy gene expression profiling of sorted cell subpopulations from breast cancer PDX model tissue
Source: PLoS One. 2020 Sep 10;15(9):e0238594. doi: 10.1371/journal.pone.0238594 (PMC7482927; doi:10.1371/journal.pone.0238594)
Supplement: S1 File — (DOCX) [file pone.0238594.s001.docx]

The supporting information contains additional data supporting our conclusions as well as material to facilitate comparison to other work. Towards the latter point Table 10 contains the respective lists of genes extracted from literature cited in S4 to S16 Figs and S11 to S13 Tables contain the three lists resulting from each comparison between marker high and marker low populations for BRC12/CD184, BRC13/CD49f and BRC13/CD133.
