# Supplementary material for: High accuracy gene expression profiling of sorted cell subpopulations from breast cancer PDX model tissue
Source: PLoS One. 2020 Sep 10;15(9):e0238594. doi: 10.1371/journal.pone.0238594 (PMC7482927; doi:10.1371/journal.pone.0238594)
Supplement: S1 Fig — Plots represent sample before (left) and after (right, overlay of 4 samples) sorting with CD49f and CD133, resp. These plots are representative of all replicate tumors. (PDF) [file pone.0238594.s002.pdf]

Pre-Sort Total Tumor Suspension

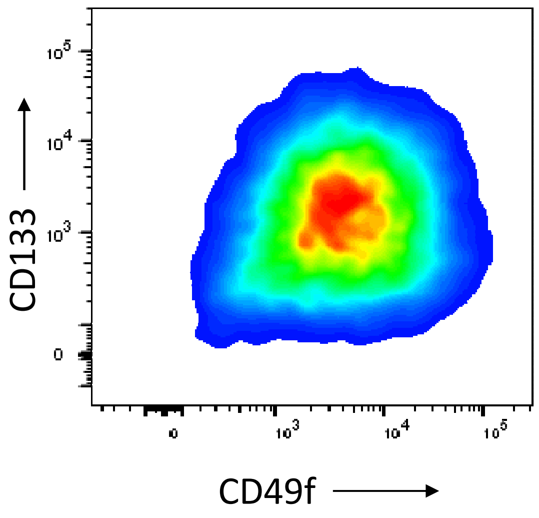

Overlay of Post-Sort Samples for Expression Analysis

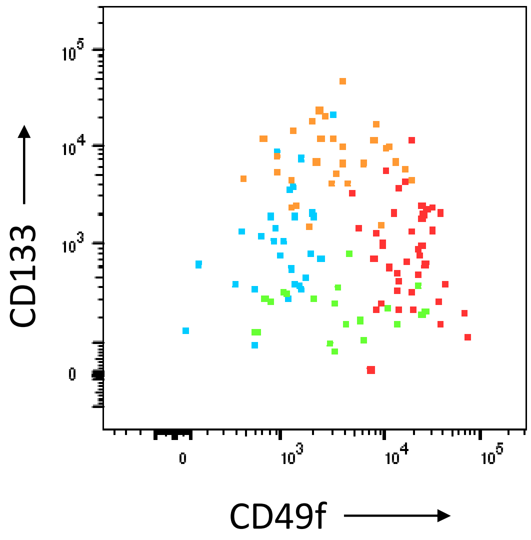

	Sample Name	Subset Name	Count
	T290_CD133 Low post_016.fcs	Tumor Cells	20.0
	T290_CD133 High post_017.fcs	Tumor Cells	30.0
	T290_CD49f Low post_014.fcs	Tumor Cells	29.0
	T290_CD49f High post_015.fcs	Tumor Cells	41.0
