# Supplementary material for: High accuracy gene expression profiling of sorted cell subpopulations from breast cancer PDX model tissue
Source: PLoS One. 2020 Sep 10;15(9):e0238594. doi: 10.1371/journal.pone.0238594 (PMC7482927; doi:10.1371/journal.pone.0238594)
Supplement: S2 Fig — Genes encoding the surface markers which were used to sort the populations (CD184/CXCR4, CD49f/ITGA6, CD133/Prom1) were found to be differentially expressed with high statistical relevance in each experiment. CD184 (left) data is from a prior publication [12]. The horizontal line marks FDR = 0.1. (PDF) [file pone.0238594.s003.pdf]

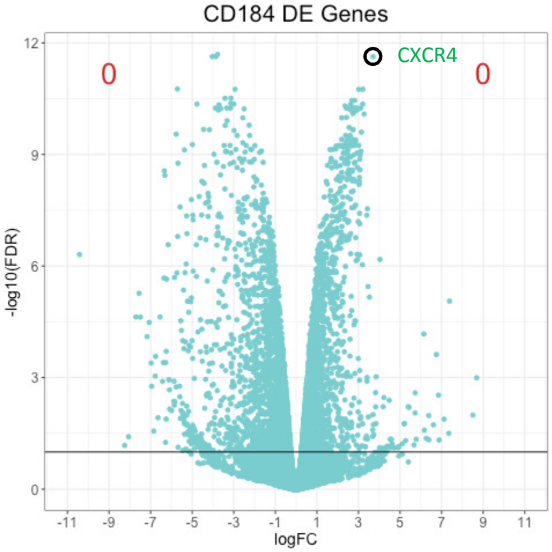

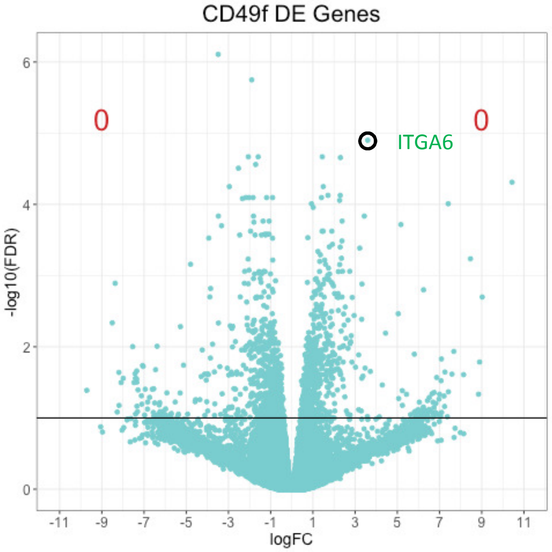

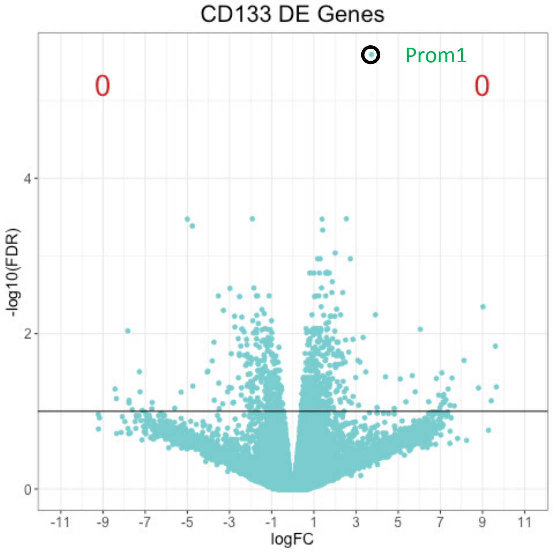

	gene	logFC	FDR	x-fold
1	KRT15	-3.8	2.0E-12	13.7
2	*CXCR4*	3.7	2.3E-12	13.0
3	KRT17	-3.9	2.3E-12	14.6
4	KRT81	-4.0	2.3E-12	16.4
5	AHNAK	-5.7	1.7E-11	52.4

	gene	logFC	FDR	x-fold
1	NTRK2	-3.5	7.8E-07	11.2
2	HK2	-1.9	1.8E-06	3.7
3	*ITGA6*	3.6	1.3E-05	12.1
4	ALPL	-2.1	2.1E-05	4.2
5	CPT1A	1.4	2.1E-05	2.7

	gene	logFC	FDR	x-fold
1	*PROM1*	3.7	2.6E-06	13.3
2	ZNF385C	-1.9	3.3E-04	3.8
3	*CXCL12*	2.5	3.3E-04	5.8
4	COL2A1	-5.0	3.4E-04	32.0
5	CNN2	1.4	3.4E-04	2.6
