# Supplementary material for: High accuracy gene expression profiling of sorted cell subpopulations from breast cancer PDX model tissue
Source: PLoS One. 2020 Sep 10;15(9):e0238594. doi: 10.1371/journal.pone.0238594 (PMC7482927; doi:10.1371/journal.pone.0238594)
Supplement: S3 Fig — Even up to very high FDR values the number of genes assessed to be different is very low for all comparisons between samples during the sample prep phase while the number of genes differentially expressed between sorted subpopulations grows continuously with increasing FDR. (PDF) [file pone.0238594.s004.pdf]

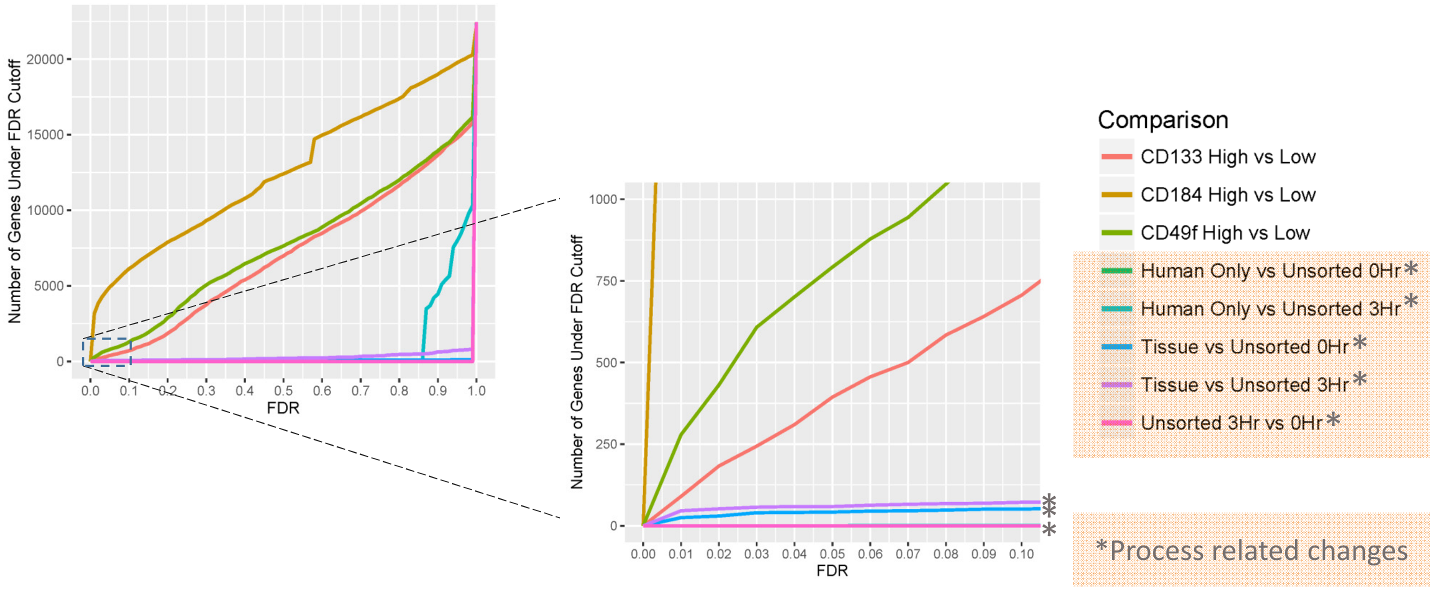
Number of Genes Under FDR Cutoff
20000
15000
10000
5000
0
0.0
0.1
0.2
0.3
0.4
0.5
0.6
0.7
0.8
0.9
1.0
FDR
Number of Genes Under FDR Cutoff
1000
750
500
250
0
0.00
0.01
0.02
0.03
0.04
0.05
0.06
0.07
0.08
0.09
0.10
FDR
Comparison
CD133 High vs Low
CD184 High vs Low
CD49f High vs Low
Human Only vs Unsorted 0Hr*
Human Only vs Unsorted 3Hr*
Tissue vs Unsorted 0Hr*
Tissue vs Unsorted 3Hr*
Unsorted 3Hr vs 0Hr*
*Process related changes
