# Supplementary figures and images for: High accuracy gene expression profiling of sorted cell subpopulations from breast cancer PDX model tissue
Source: PLoS One. 2020 Sep 10;15(9):e0238594. doi: 10.1371/journal.pone.0238594 (PMC7482927; doi:10.1371/journal.pone.0238594)

-log FDR

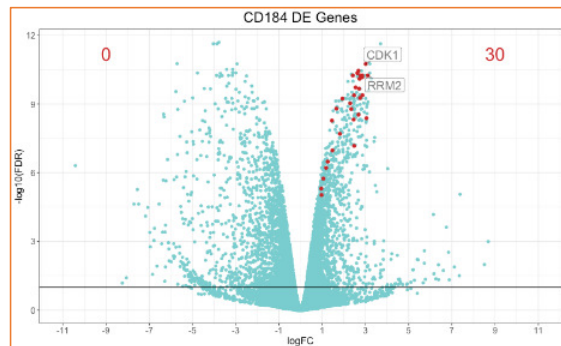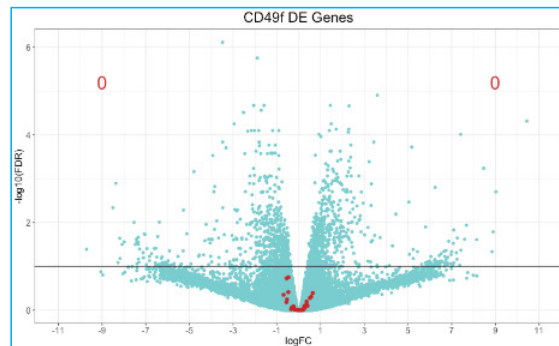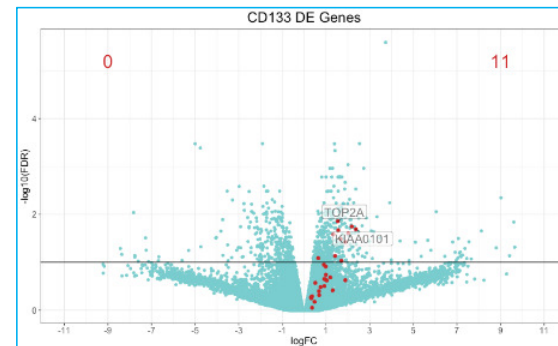

log FC

-log FDR

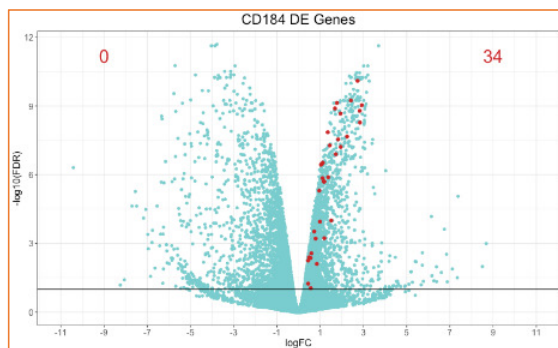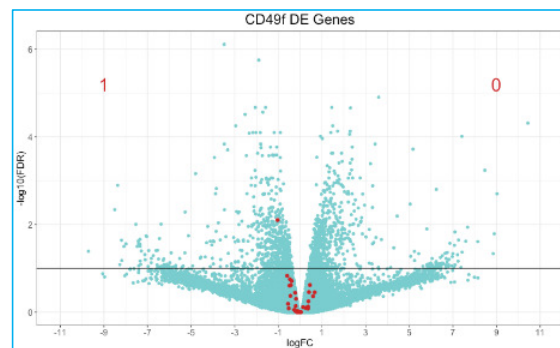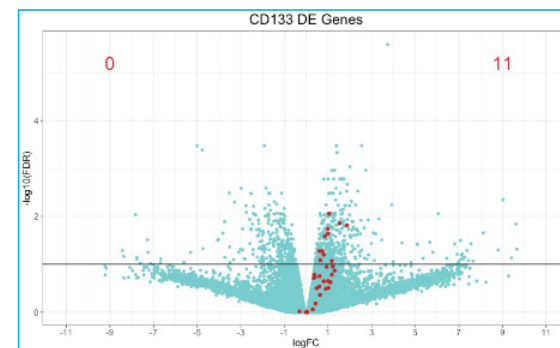

log FC

Supplement: S4 Fig — Overlap of a proliferative gene signature from histologically normal breast tissue (30 genes, [53], top; same as in Fig 5) and "hallmark E2F target genes" (34 genes, [54], bottom) with resp. gene sets within this publication are shown in volcano plots Numbers provided are genes above FDR cutoff of 0.1 up (right) or down (left) regulated. (PDF) [file pone.0238594.s005.pdf]

Chen2010prolif

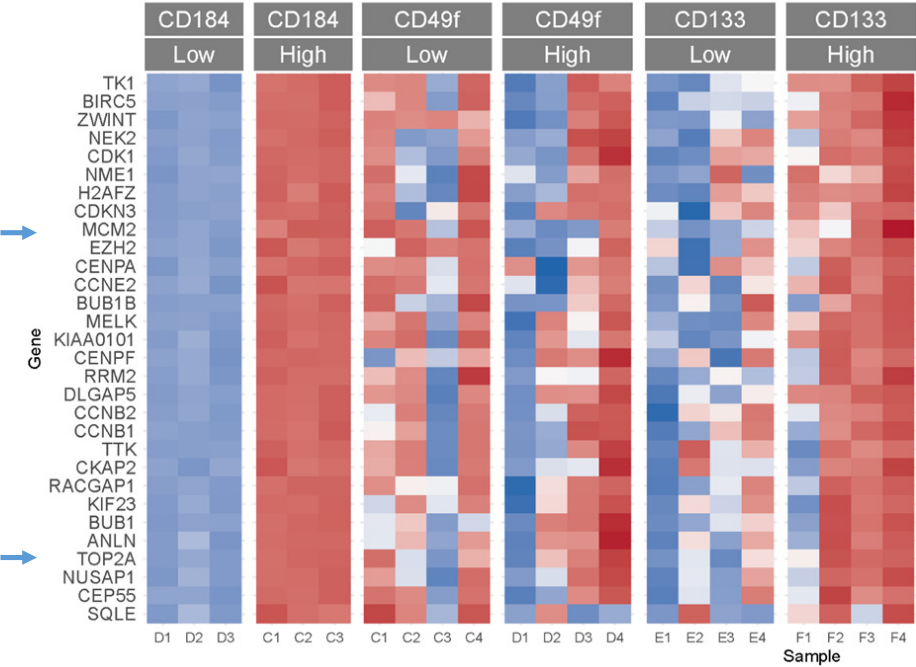

Smith2015\_E2F

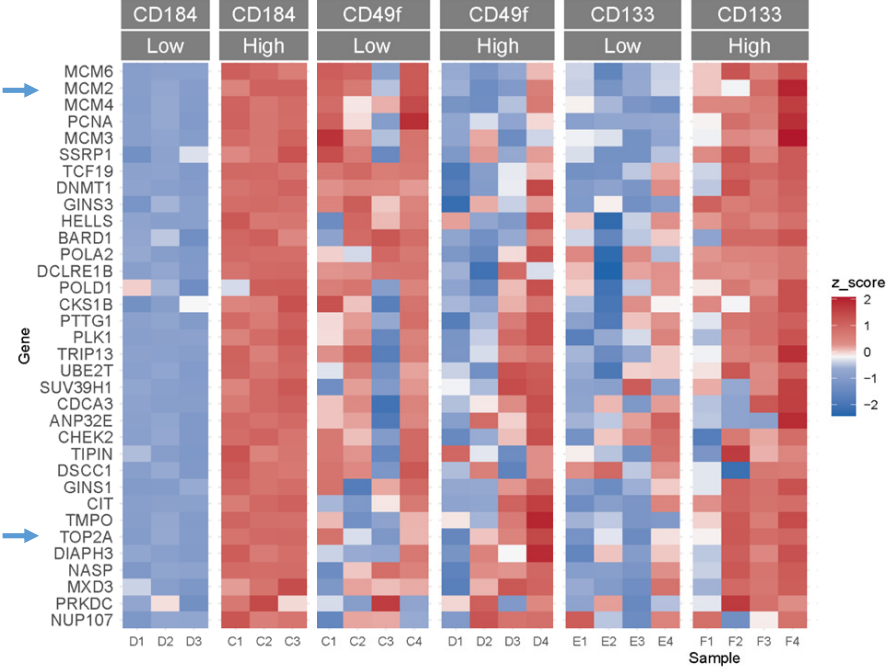

Supplement: S5 Fig — Overlap of a proliferative gene signature from histologically normal breast tissue (30 genes, [53], left; same as in Fig 5) and "hallmark E2F target genes" (34 genes, [54], right) with resp. gene sets within this publication are shown in heatmaps. 2 genes (TOP2A and MCM2, see arrows) overlap between the signatures. (PDF) [file pone.0238594.s006.pdf]

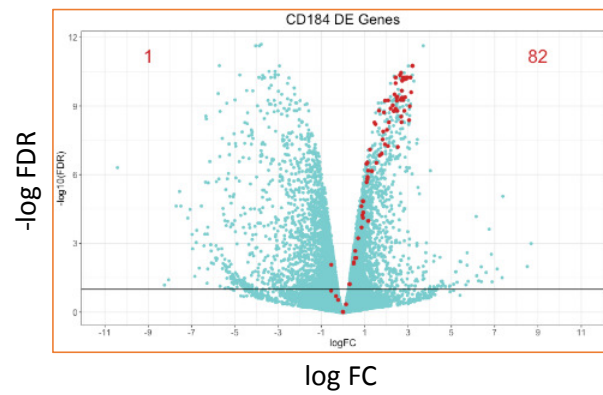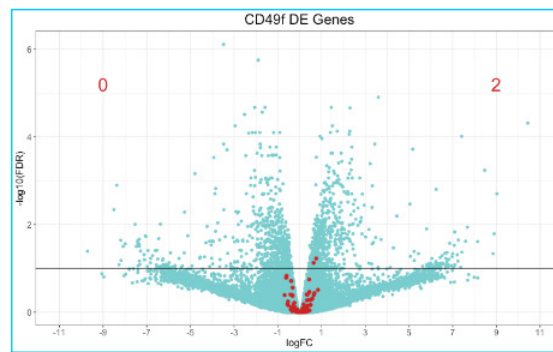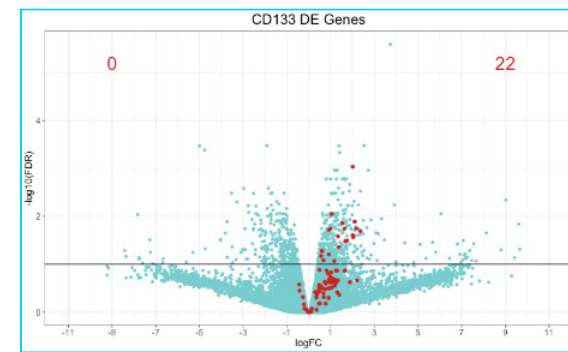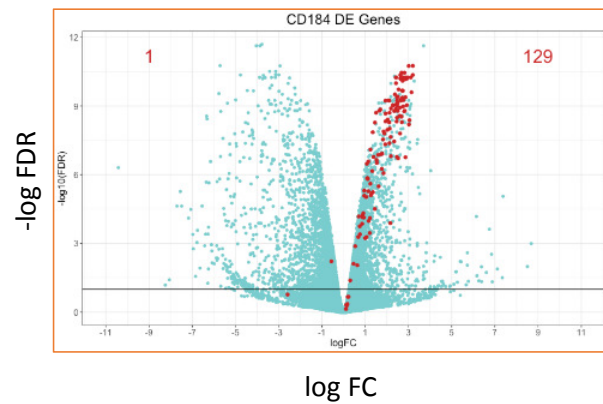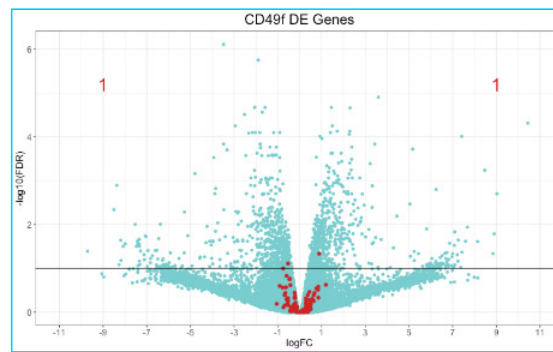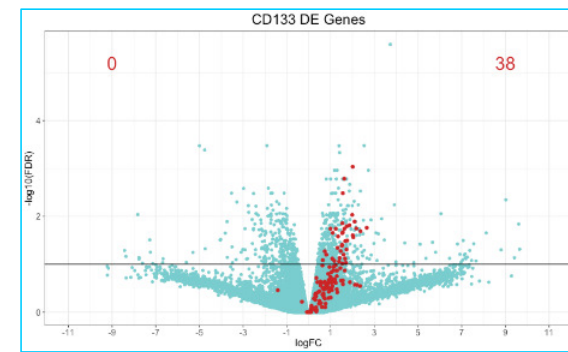

Supplement: S6 Fig — Overlap of a stem cell gene signature [55], (top/) and a genomic instability signature identified in glioma [56] (bottom) are shown in volcano plots. (PDF) [file pone.0238594.s007.pdf]

-log FDR

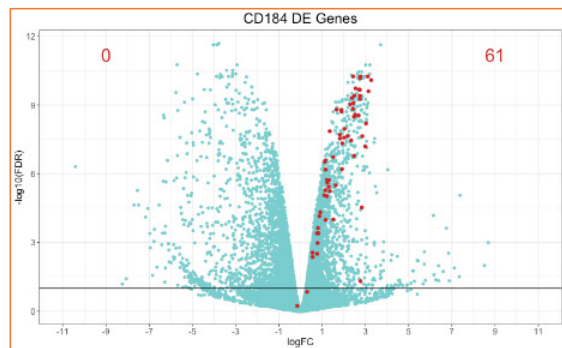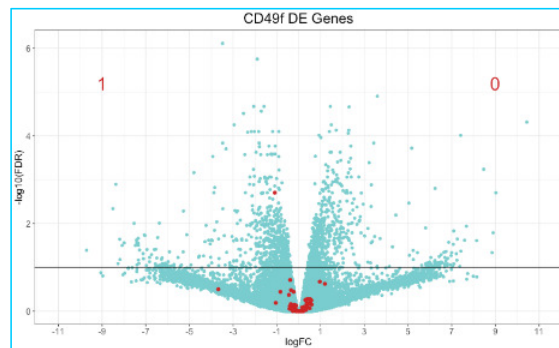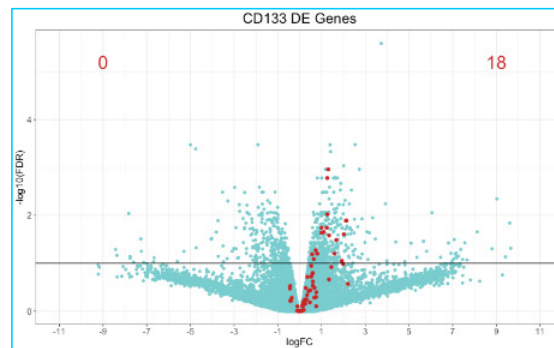

log FC

-log FDR

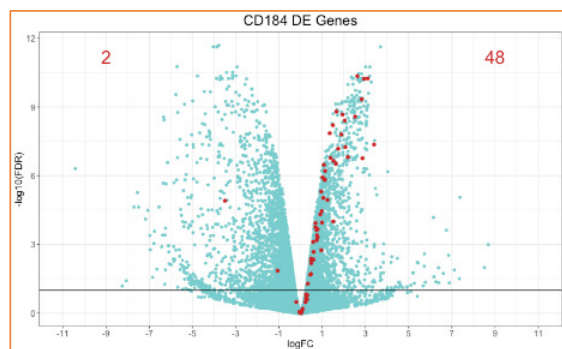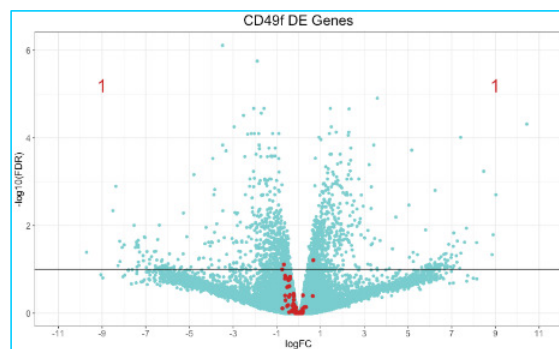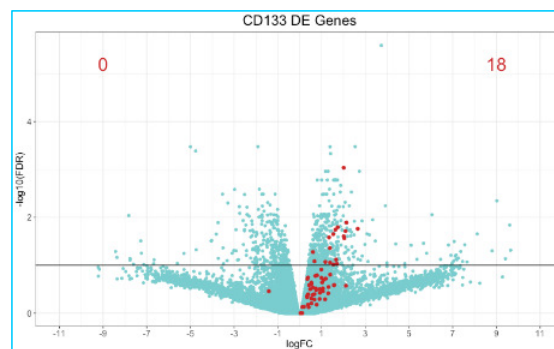

log FC

Supplement: S8 Fig — Overlap of a subpopulation signature associated with poor outcome in lung adenocarcinoma [57] (top) and a signature associated with DNA damage response (DDR) and resistance to PARP inhibition [58] (bottom) are shown in volcano plots. (PDF) [file pone.0238594.s009.pdf]

Min2015G64

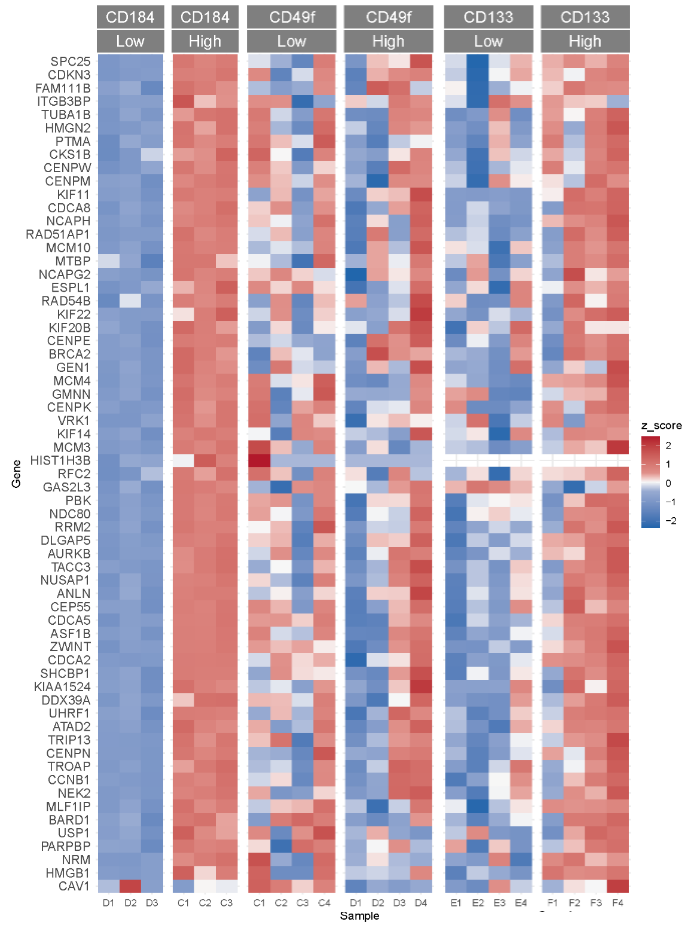

Hassan2017PARPi

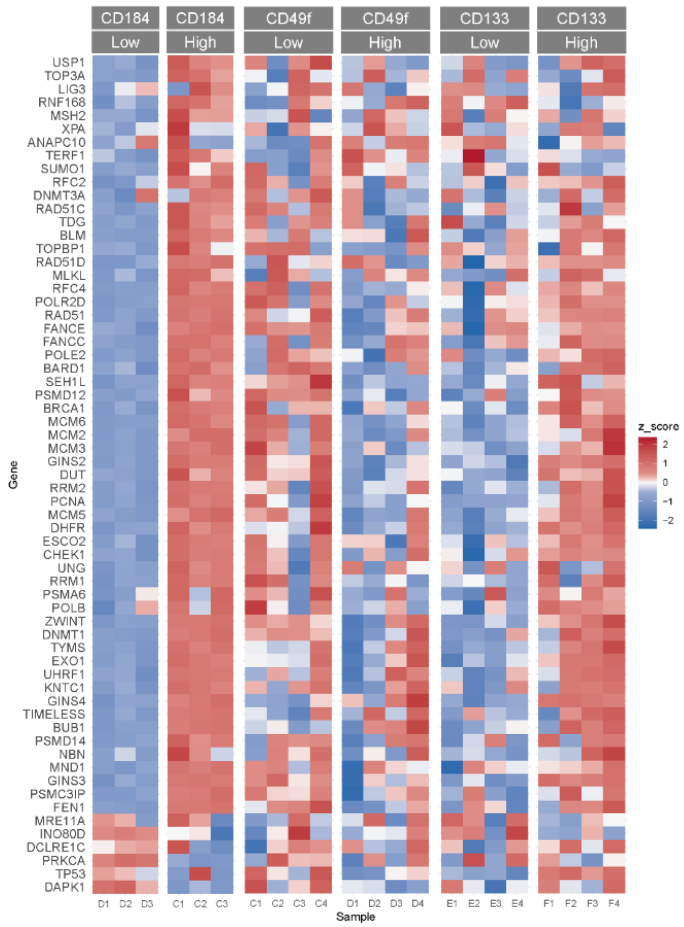

Supplement: S9 Fig — Overlap of a subpopulation signature associated with poor outcome in lung adenocarcinoma [57] (left) and a signature associated with DNA damage response (DDR) and resistance to PARP inhibition [58] (right) are shown in heatmaps (b). (PDF) [file pone.0238594.s010.pdf]

-log FDR

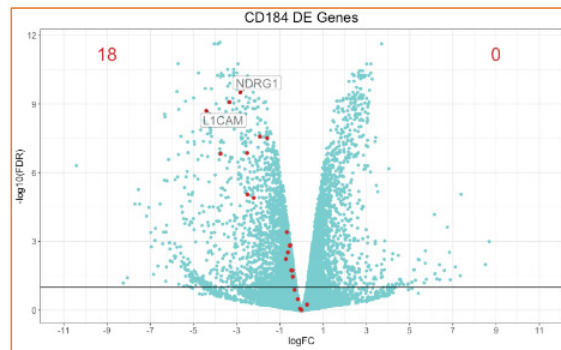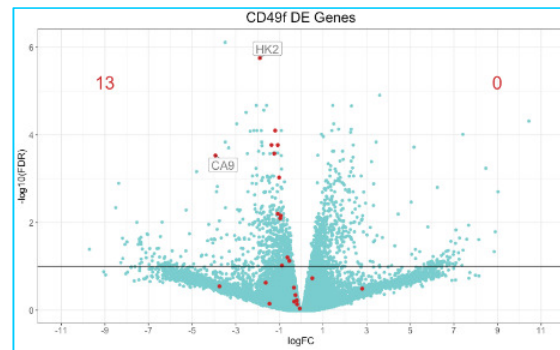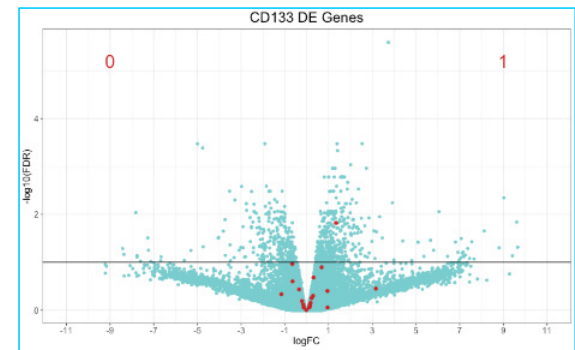

log FC

-log FDR

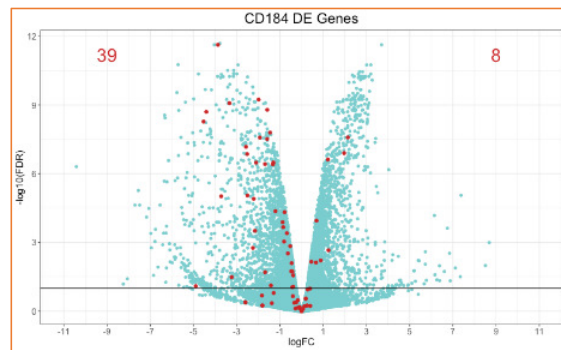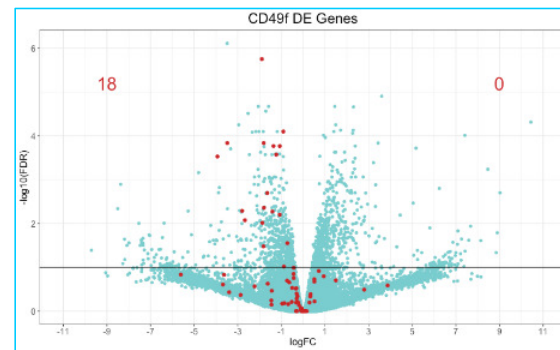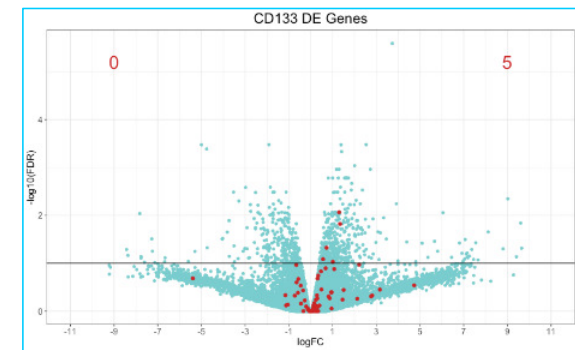

log FC

Supplement: S10 Fig — Overlap of a hypoxia signature composed from 3 publications (see Fig 5a, [34–36], top) and a larger hypoxia gene set described in [59], (bottom) are shown in volcano plots. (PDF) [file pone.0238594.s011.pdf]

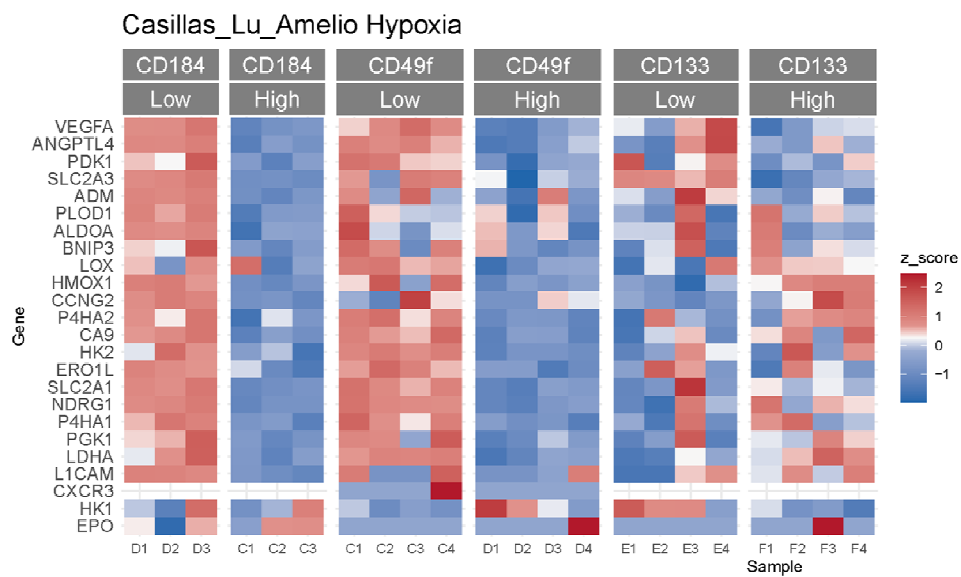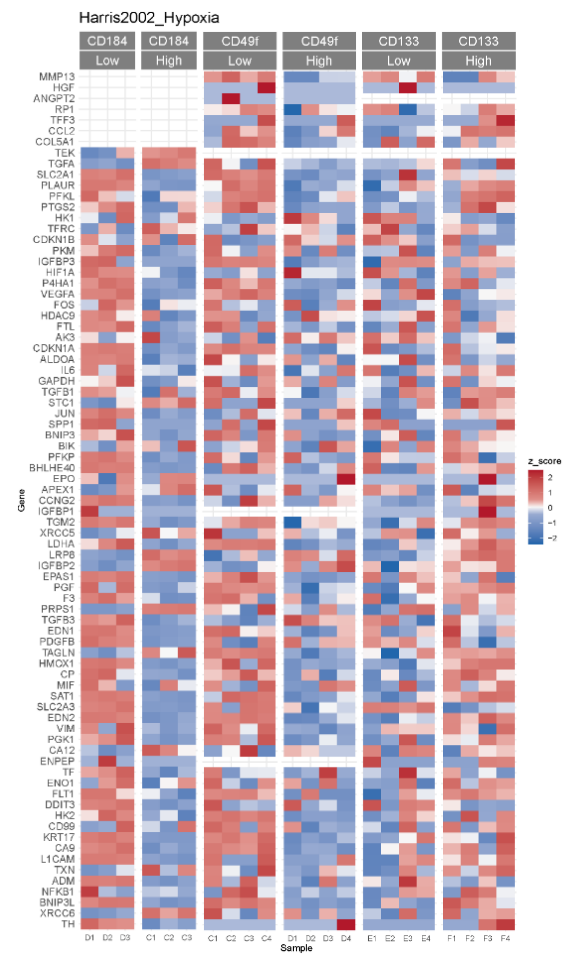

Supplement: S11 Fig — Overlap of a hypoxia signature composed from 3 publications (see Fig 5a, [34–36], left) and a larger hypoxia gene set described in [59], (right) are shown in heatmaps (b). (PDF) [file pone.0238594.s012.pdf]

-log FDR

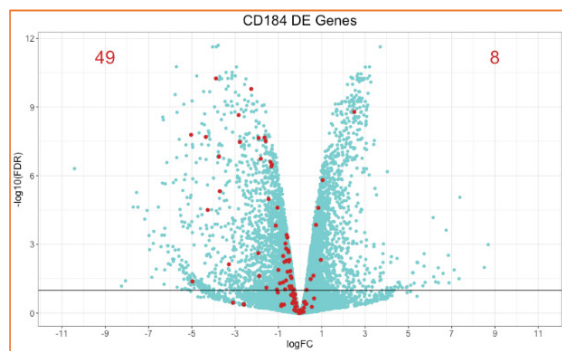

log FC

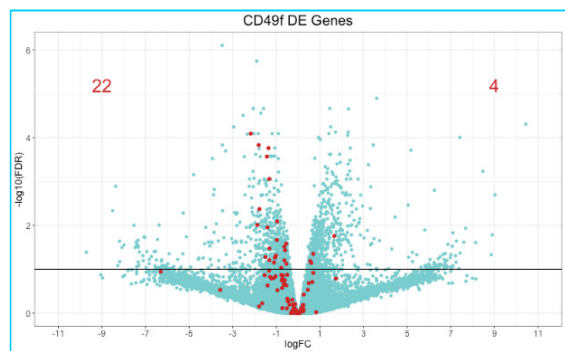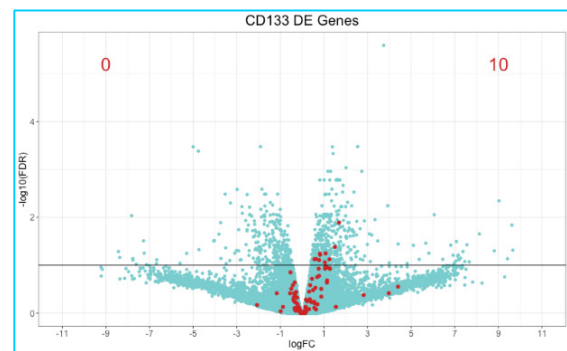

Supplement: S12 Fig — Overlap of a drug resistance signature described in lung carcinoma and melanoma [60] are shown in a volcano plot. (PDF) [file pone.0238594.s013.pdf]

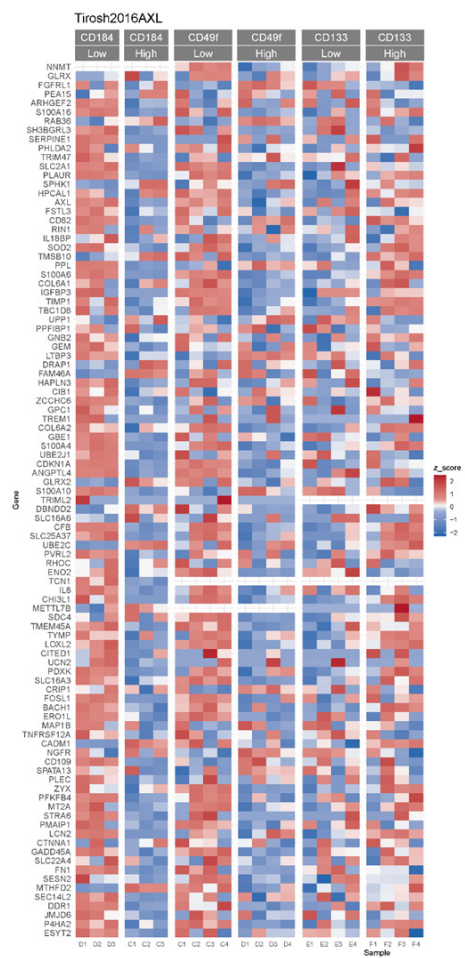

Supplement: S13 Fig — Overlap of a drug resistance signature described in lung carcinoma and melanoma [60] are shown in a heatmap. (PDF) [file pone.0238594.s014.pdf]

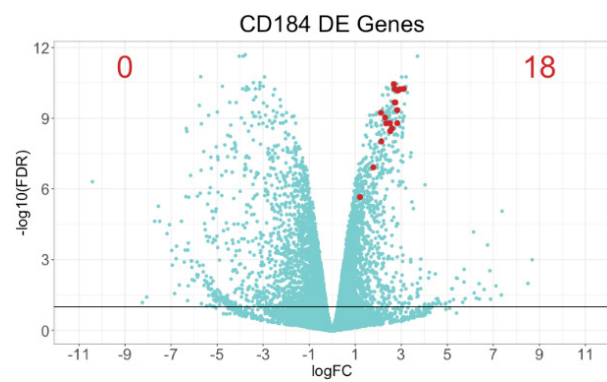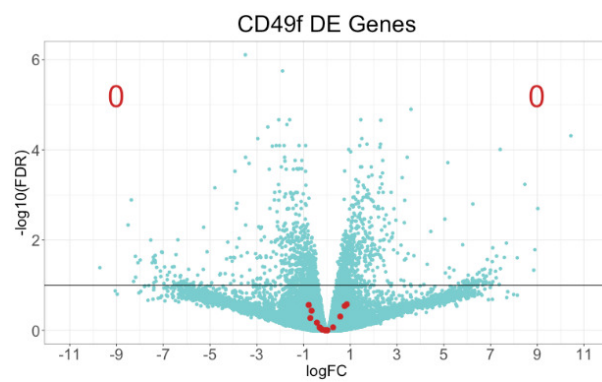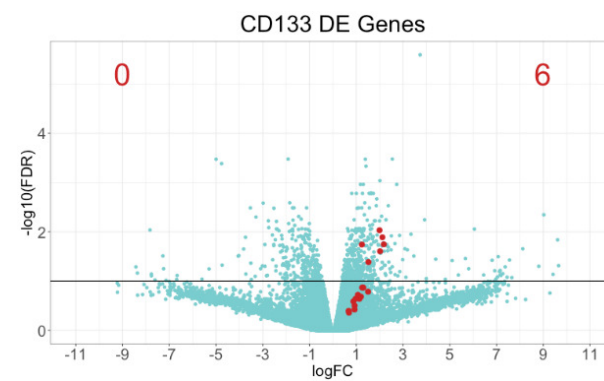

Supplement: S14 Fig — Overlap of 19 genes associated with proliferation in a sscRNASeq analysis of TNBC tissue [61]. (PDF) [file pone.0238594.s015.pdf]

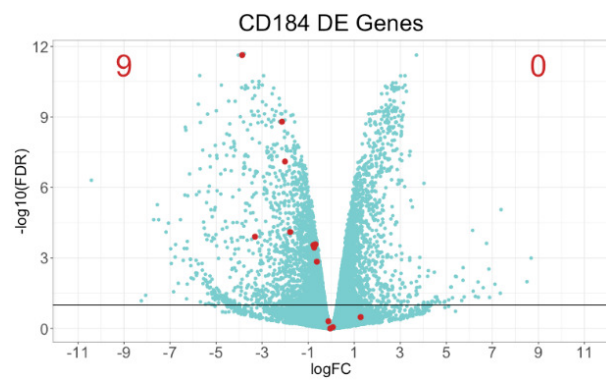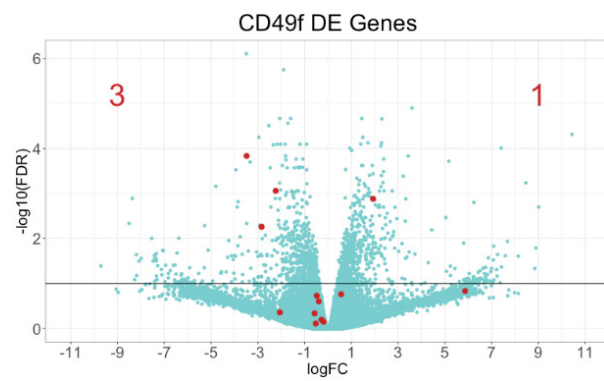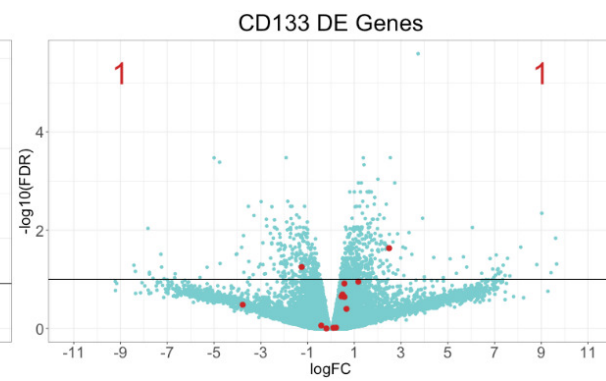

Supplement: S15 Fig — Overlap of 18 genes associated with an epithelial phenotype in a scRNASeq study on TNBC patient samples [62]. (PDF) [file pone.0238594.s016.pdf]

(a)

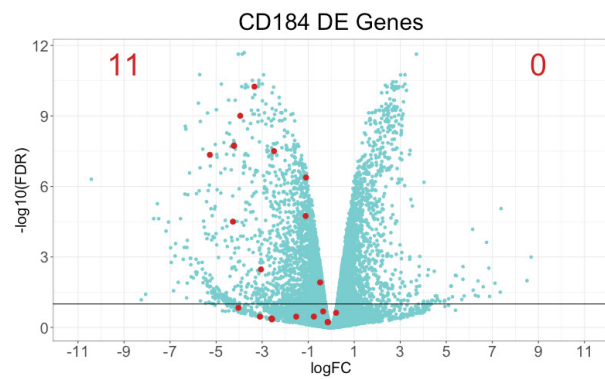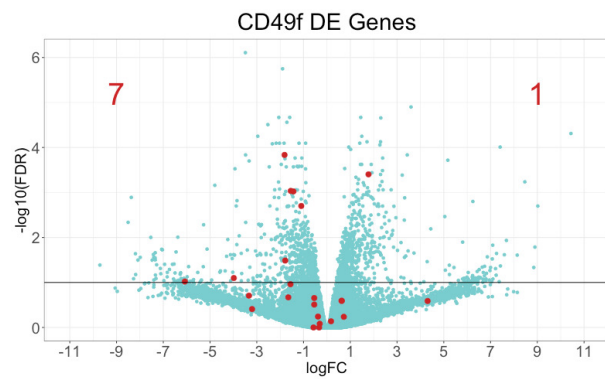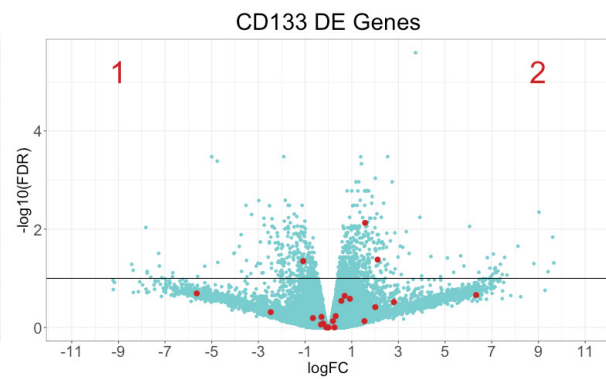

(b)

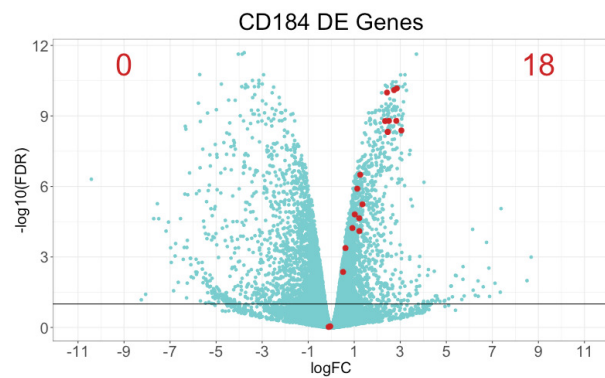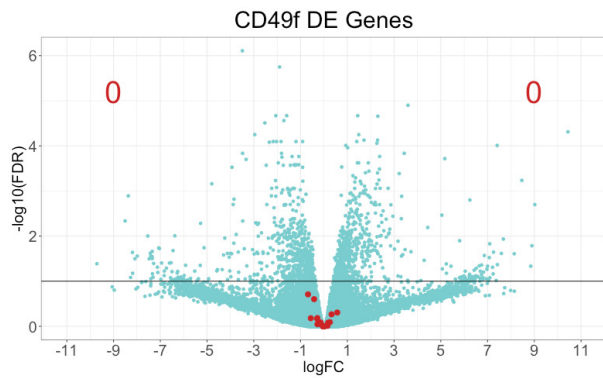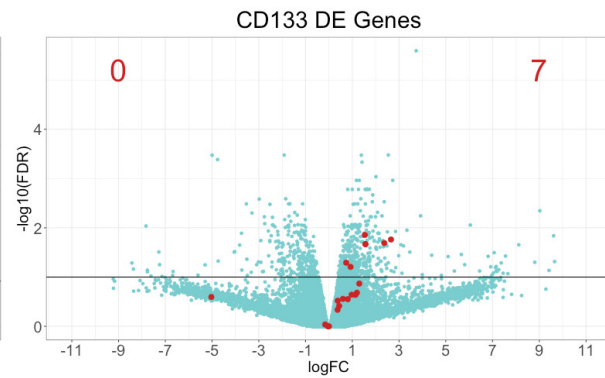

Supplement: S16 Fig — Overlap genes identified in different clusters within single cells from the same tissue in PDX models of TNBC [63] A) 24 combined genes most upregulated in clusters 1,3,6 B) 20 combined genes most upregulated in clusters 2,4. (PDF) [file pone.0238594.s017.pdf]
