# Supplementary material for: High accuracy gene expression profiling of sorted cell subpopulations from breast cancer PDX model tissue
Source: PLoS One. 2020 Sep 10;15(9):e0238594. doi: 10.1371/journal.pone.0238594 (PMC7482927; doi:10.1371/journal.pone.0238594)
Supplement: S7 Fig — Overlap of a stem cell gene signature [55], (left) and a genomic instability signature identified in glioma [56] (right) are shown in heatmaps (b). (PDF) [file pone.0238594.s008.pdf]

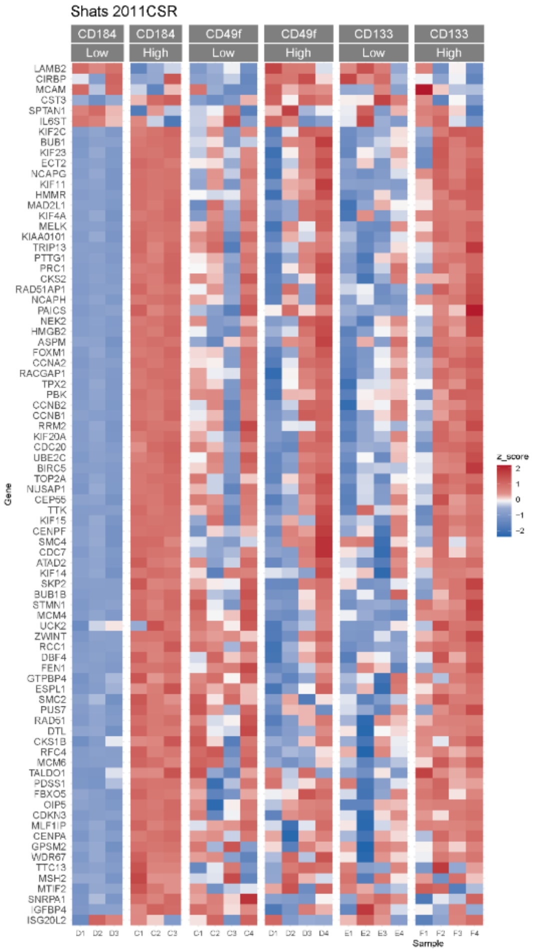
Shats 2011CSR
CD184 Low
CD184 High
CD49f Low
CD49f High
CD133 Low
CD133 High
LAMB2
CIRBP
MCAM
CST3
SPTAN1
IL6ST
KIF2C
BUB1
KIF23
ECT2
NCAPG
KIF11
HMMR
MAD2L1
KIF4A
MELK
KIAA0101
TRIP13
PTTG1
PRC1
CKS2
RAD51AP1
NCAPH
PAICS
NEK2
HMGB2
ASPM
FOXM1
CCNA2
RACGAP1
TPX2
PBK
CCNB2
CCNB1
RRM2
KIF20A
CDC20
UBE2C
BIRC5
TOP2A
NUSAP1
CEP55
TTK
KIF15
CENPF
SMC4
CDC7
ATAD2
KIF14
SKP2
BUB1B
STMN1
MCM4
UCK2
ZWINT
RCC1
DBF4
FEN1
GTPBP4
ESPL1
SMC2
PUS7
RAD51
DTL
CKS1B
RFC4
MCM6
TALDO1
PDSS1
FBXO5
OIP5
CDKN3
MLF1IP
CENPA
GPSM2
WDR67
TTC13
MSH2
MTIF2
SNRPA1
IGFBP4
ISG20L2
Gene
z_score
2
1
0
-1
-2
D1 D2 D3
C1 C2 C3
C1 C2 C3 C4
D1 D2 D3 D4
E1 E2 E3 E4
F1 F2 F3 F4
Sample

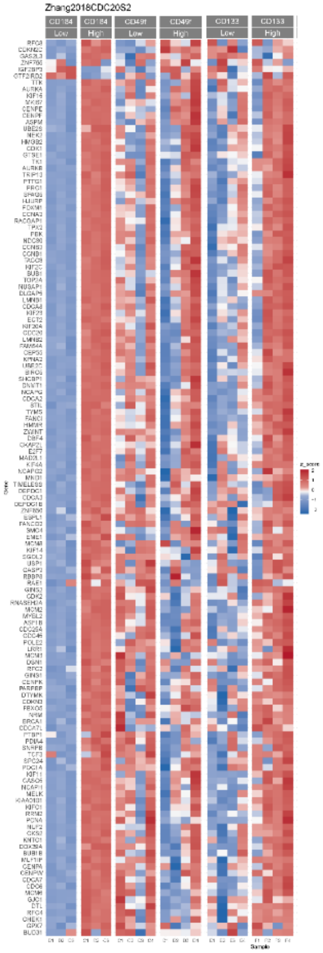
Zhang2018CDC20S2
CD184 Low
CD184 High
CD49f Low
CD49f High
CD133 Low
CD133 High
