# Supplementary material for: High accuracy gene expression profiling of sorted cell subpopulations from breast cancer PDX model tissue
Source: PLoS One. 2020 Sep 10;15(9):e0238594. doi: 10.1371/journal.pone.0238594 (PMC7482927; doi:10.1371/journal.pone.0238594)
Supplement: Attachment — Submitted filename: Response to Reviewers.docx [file pone.0238594.s031.docx]

Journal Requirements:

When submitting your revision, we need you to address these additional requirements.

1. Please ensure that your manuscript meets PLOS ONE's style requirements, including those for file naming. The PLOS ONE style templates can be found at

<https://journals.plos.org/plosone/s/file?id=wjVg/PLOSOne_formatting_sample_main_body.pdf> and

<https://journals.plos.org/plosone/s/file?id=ba62/PLOSOne_formatting_sample_title_authors_affiliations.pdf>

We have tried to adhere to the specifications for the figure files but as some metrics are not completely unambiguous would request some guidance or assistance in case there are discrepancies.

2. At this time, we request that you  please report additional details in your Methods section regarding animal care, as per our editorial guidelines:

(1) Please provide details of animal welfare (e.g., shelter, food, water, environmental enrichment)

(2) Please include the method of euthanasia

(3) Please describe the care received by the animals, including the frequency of monitoring and the criteria used to assess animal health and well-being during the course of the tumor study.

Thank you for your attention to these requests.

Changes were made to the methods section to include the details requested. We opted to omit the list of clinical signs of indications of morbidity we use (below) for conciseness but would be happy to include this as well if requested:

The following clinical signs are indications of morbidity:

1.	Persistent anorexia or dehydration

2.	Consistent or rapid weight loss of 20% maintained for 72 hours.

3.	Unable to maintain an upright posture or to move.

4.	Muscle atrophy or emaciation.

5.	Lethargy or failure to respond to gentle stimuli.

6.	Hyperthermia.

7.	Unconsciousness or coma.

8.	Bloodstained or mucropurulent discharge from any orifice.

9.	Labored respiration – particularly if accompanied by nasal discharge or cyanosis.

10.	Anemia.

11.	Significant abdominal distension.

12.	Incontinence or prolonged diarrhea.

13.	Dragging a limb or having trouble ambulating.

3. Thank you for including your ethics statement: 'All animal studies were performed under an Institutional Animal Care and Use Committee–approved protocol.'

(a) Please amend your current ethics statement to include the full name of the ethics committee/institutional review board(s) that approved your specific study.

(b) Once you have amended this/these statement(s) in the Methods section of the manuscript, please add the same text to the “Ethics Statement” field of the submission form (via “Edit Submission”).

For additional information about PLOS ONE ethical requirements for human subjects research, please refer to <http://journals.plos.org/plosone/s/submission-guidelines#loc-human-subjects-research>.

We believe this does not apply to our study since we used PDX models commercially available from Jackson Labs and an IRB was not required. Please advise if this is in conflict with your interpretation of this requirement.

4. Please note that PLOS does not permit references to “data not shown.” Authors should provide the relevant data within the manuscript, the Supporting Information files, or in a public repository. If the data are not a core part of the research study being presented, we ask that authors remove any references to these data. Done

5. Please provide the product number and any lot numbers of the primary antibodies purchased from chemical companies for your study.

A table has been added in the supplemental material listing this information.

6. To comply with PLOS ONE submission guidelines, in your Methods section, please provide additional information regarding your statistical analyses. For more information on PLOS ONE's expectations for statistical reporting, please see <https://journals.plos.org/plosone/s/submission-guidelines.#loc-statistical-reporting>.

The Methods section has been augmented with the respective information

7. We note that you have indicated that data from this study are available upon request. PLOS only allows data to be available upon request if there are legal or ethical restrictions on sharing data publicly. For more information on unacceptable data access restrictions, please see <http://journals.plos.org/plosone/s/data-availability#loc-unacceptable-data-access-restrictions>.

The respective data will be made public by uploading the entire RNASeq data set to an appropriate public server (see below).

In your revised cover letter, please address the following prompts:

a) If there are ethical or legal restrictions on sharing a de-identified data set, please explain them in detail (e.g., data contain potentially sensitive information, data are owned by a third-party organization, etc.) and who has imposed them (e.g., an ethics committee). Please also provide contact information for a data access committee, ethics committee, or other institutional body to which data requests may be sent.
N/A

b) If there are no restrictions, please upload the minimal anonymized data set necessary to replicate your study findings as either Supporting Information files or to a stable, public repository and provide us with the relevant URLs, DOIs, or accession numbers. For a list of acceptable repositories, please see <http://journals.plos.org/plosone/s/data-availability#loc-recommended-repositories>.
In process; we will submit confirmation as soon as available.

We will update your Data Availability statement on your behalf to reflect the information you provide.

8. Thank you for stating the following in the Competing Interests section:

WP, ES, FT, WSD and RB are employees of BD and FH and MF were BD employees
at the time the work was carried out.

We note that one or more of the authors are employed by a commercial company: BD Technologies and Innovation,
(I have corrected the exact wording)

a. Please provide an amended Funding Statement declaring this commercial affiliation, as well as a statement regarding the Role of Funders in your study. If the funding organization did not play a role in the study design, data collection and analysis, decision to publish, or preparation of the manuscript and only provided financial support in the form of authors' salaries and/or research materials, please review your statements relating to the author contributions, and ensure you have specifically and accurately indicated the role(s) that these authors had in your study. You can update author roles in the Author Contributions section of the online submission form.
(I have completed using other company papers as guidance)

Please also include the following statement within your amended Funding Statement.

“The funder provided support in the form of salaries for authors [insert relevant initials], but did not have any additional role in the study design, data collection and analysis, decision to publish, or preparation of the manuscript. The specific roles of these authors are articulated in the ‘author contributions’ section.”

If your commercial affiliation did play a role in your study, please state and explain this role within your updated Funding Statement.

 (I have completed using other company papers as guidance)

b. Please also provide an updated Competing Interests Statement declaring this commercial affiliation along with any other relevant declarations relating to employment, consultancy, patents, products in development, or marketed products, etc.

Within your Competing Interests Statement, please confirm that this commercial affiliation does not alter your adherence to all PLOS ONE policies on sharing data and materials by including the following statement: "This does not alter our adherence to  PLOS ONE policies on sharing data and materials.” (as detailed online in our guide for authors [http://journals.plos.org/plosone/s/competing-interests](about:blank)) . If this adherence statement is not accurate and  there are restrictions on sharing of data and/or materials, please state these. Please note that we cannot proceed with consideration of your article until this information has been declared.

 (I have completed using other company papers as guidance)

Please include both an updated Funding Statement and Competing Interests Statement in your cover letter. We will change the online submission form on your behalf.

Please know it is PLOS ONE policy for corresponding authors to declare, on behalf of all authors, all potential competing interests for the purposes of transparency. PLOS defines a competing interest as anything that interferes with, or could reasonably be perceived as interfering with, the full and objective presentation, peer review, editorial decision-making, or publication of research or non-research articles submitted to one of the journals. Competing interests can be financial or non-financial, professional, or personal. Competing interests can arise in relationship to an organization or another person. Please follow this link to our website for more details on competing interests: [http://journals.plos.org/plosone/s/competing-interests](about:blank)

# Response to Reviewers

5. Review Comments to the Author

Please use the space provided to explain your answers to the questions above. You may also include additional comments for the author, including concerns about dual publication, research ethics, or publication ethics. (Please upload your review as an attachment if it exceeds 20,000 characters)

Reviewer #1: In “High accuracy gene expression profiling of sorted cell subpopulations from breast cancer PDX model tissue”, Porter et al study the influence of fluorescence activated cell sorting (FACS) on transcriptional profiling and investigate the presence of multiple different subpopulations within the same breast cancer model tissue.” The manuscript seeks to understand whether there are consistent expression features of cell subpopulations in tumors, by using a xenograft model system. This is an important question for the field. While the techniques used are not at the forefront (scRNA-seq would be preferable), the concept of the work is sound. I have a few major concerns and some minor critiques.

Major

Line 180: The authors should provide a formal gene set analysis of the genes that were DE in both experiments, as well as for the DE genes unique to individual experiments. The analysis provided in lines 192-203 is relevant to this but is too inexact to be evaluated.

A gene set analysis in Enrichr was performed and supplied in the supplemental material along with a description in the results section.

The authors have a sizable number of RNA-seq replicates (line 132 suggests there are 32 RNA-seq datasets, though it is difficult to tell from the text) both across and within xenografts. However, there is little discussion of the robustness of the DE analysis across and within mice. This high number of replicates is the major strength of the work, so comparisons of replicates should be explicit.

*Clarifying language was added in line 132 (caption Fig.1) and 137.*

The authors should describe how many cells were in each of the sorted populations. The proportions of cell populations that appear within xenograft ecosystems has been studied in pubs such as [Cell 167, 260–274.e22 (2016); Nat Comms 5, 5871 (2014); Sci Rep 8, 17937 (2018)] and may be relevant for tumor growth. Comments on this topic would strengthen the work.

*We appreciate this helpful suggestion. We provided the ratio of the respective supopulations in the supplemental data (S2) and added a paragraph about various forms of ITH (genotypic and phenotypic) and reports of outcomes of mixing cells with defined genotypic and or phenotypic characteristics. Since the primary aim of our work is in providing the most efficient path towards future functional studies of subpopulation interactions we refrain from drawing conclusions on the dynamic involved in our work for which we believe more than one time point would be required.*

On a related point, are there published scRNA TNBC datasets available that the authors can compare to? Comparisons to such data would clarify the robustness of the observed effects.

*We surveyed the literature specifically to address this point. There is no single repository with standard search protocols without which an exhaustive search would exceed our resources. We were however able to identify multiple examples of significant overlap between DEGs detected in scRNASeq data from TNBC and our gene lists. While we cannot exclude the possibility that additional relevant profiles exist we added three representative examples as supplemental figures (S9-S11) and edited the discussion accordingly.*

*Additionally we would like to point out that the gene set identified by Hassan et al. 2017 (see Fig. S6) in our original manuscript which correlates strongly with our data was identified in TNBC, albeit based on regulation within whole populations of cells.*

Minor

Line 146: Please clarify that the references indicate the genes’ IEG status. This is not apparent from the table 1 caption.

*The header of the table was changed to state “Reference identifying gene as IEG” as clarification.*

Line 182: “120 of the 121 genes in common were qualitatively correlated (i.e. identical direction of change) and on a quantitative level the correlation coefficient was R= - 0.85” : The first and second parts of the sentence are stated inconsistently. If the direction of change is identical then the correlation coefficient should be positive. Rephrase.

*We appreciate pointing out this error and have changed the wording to clearly state that it was an inverse correlation.*

Some aspects of the article are out of date. E.g. Ref 12 lists a publication from 2017 as “in press.”

*We have corrected the identified publication. Additionally we have replaced previous reference #9 with a peer reviewed publication by the same authors on the topic (previous reference was a pre-publication in Biorxiv).*

Reviewer #2: Porter et al. have submitted a will written report investigating intratumoral heterogeneity of the transcriptome within solid human tumors by isolation of distinct cancer cell populations by FACS. Across tumor cases and replicates the authors identified immediate early gene changes, primarily due to tumor dissociation, that agreed to a high degree with previous publications. Cell populations were sorted based on high/low expression of the cell surface markers CD49f, CD133, and CD184, which were then analyzed by RNAseq. Differentially expressed genes were identified between populations indicating that each population may have distinct roles within the tumors. Overall, the experiments were designed well with proper controls, and this research will likely pave the way for more functional assays in the future (e.g. therapeutic response). I have no major concerns with the manuscript and have only a few minor comments/suggestions.

- In the Introduction I would suggest adding a brief description of the biological roles of CD49f, CD133, and CD184 and/or the types of cancer cell populations they have previously been used to isolate and define.

*A passage has been added in the introduction including additional references to address this.*

- For whole fresh tumor and the “bulk” dissociated cell population, what was the degree to mouse stromal (fibroblast, endothelial, and immune cell) cell infiltration? This can often be 10-50% in solid tumors. Did this have any effect on the RNAseq analysis for these samples?

*We added the information in the results. Both models investigated by us so far contained 15% mouse cells which were excluded from the RNASeq analysis (in silico from the bulkk analysis, physically for “human only” and marker sorted cell poopulations) and to date we have not investigated differing models which would allow us to draw any conclusions in that regard.*
